# Viral Hepatitis A to E in South Mediterranean Countries

**DOI:** 10.4084/MJHID.2010.001

**Published:** 2010-02-10

**Authors:** Sanaa M. Kamal, Sara Mahmoud, Tamer Hafez, Runia EL-Fouly

**Affiliations:** Department of Tropical Medicine, Gastroenterology and Liver Disease, Ain Shams University, Cairo, Egypt

## Abstract

Viral hepatitis represents an important health problem in the South Mediterranean countries, Egypt, Libya, Tunisia, Algeria and Morocco. Emerging natural history and epidemiological information reveal differences in the overall epidemiology, risk factors and modes of transmission of viral hepatitis A, B, C, D, E infections in the South Mediterranean region. The differences in the in incidence and prevalence of viral hepatitis across North African countries is attributed to variations in health care and sanitation standards, risk factors and immunization strategies. The active continuous population movement through travel, tourism and migration from and to the South Mediterranean countries contribute to the spread of infections due to hepatitis viruses across borders leading to outbreaks and emergence of new patterns of infection or introduction of uncommon genotypes in other countries, particularly in Europe.

## Introduction:

In There are some similarities but also there substantial di€erences between South Mediterranean countries. The epidemiology of viral hepatitis in south Mediterranean countries is dynamic and affected by many factors including hygiene, socioeconomic status and vaccination coverage. For example, hepatitis B (HBV) 1,2 and hepatitis C (HCV) viruses 3 have a high tendency to persist and establish chronic hepatitis with long-term sequels. HBV- and HCV-related chronic hepatitis is considered the main cause of cirrhosis, hepatocellular carcinoma (HCC) and liver transplantation in the region^2^,^3^. While HCV related liver disease represents a huge health and economic problem in Egypt, HCV is not considered a public health problem in Morocco. In this review, we will explore the epidemiology, patterns and trends of viral hepatitis in the South Mediterranean countries namely, Egypt, Libya, Tunisia, Algeria and Morocco.

## Hepatitis A Virus (HAV) Infection

HAV still represents a public health problem in the South Mediterranean region which is considered an area with high HAV prevalence.^4^ HAV is a frequent travel acquired infection that has caused several outbreaks among European tourists.^5^,^6^,^7^,^8^,^9^ HAV infection is largely acquired through fecal-oral transmission.^5^ Consumption of contaminated food or raw sea food or drinking contaminated water represents the main routes of transmission in the South Mediterranean region. HAV is a resilient virus that survives for prolonged periods in food and drink.^7^,^8^ HAV infection in South Mediterranean countries has been characterized with a lifetime risk of infection greater than 90%. Most infections occur in early childhood and those infected may not experience any noticeable symptoms. Epidemics are uncommon because older children and adults are generally immune. Disease rates in these areas are low and outbreaks are rare. Recently, a shift from high to intermediate HAV endemicity patterns is observed in countries with transitional economies such as Egypt, Tunisia and Morocco. In these countries, the improvement of economic and sanitary conditions has been associated with decline in early childhood HAV infections that started to occur in older age groups. Immunization against HAV is not incorporated in the routine childhood immunization programs in any of the countries of the region.

### HAV in Egypt:

HAV is endemic in Egypt and represents a major cause of acute viral hepatitis being responsible for more than half of acute s hepatitis cases. HAV genotype IB is the prevalent genotype in Egypt. Person-to-person transmission, consumption of food contaminated in fields or through infected food handlers is very common as well as consumption of contaminated mollusks (clams, oysters) and unsafe drinking water represent the major routes of HAV transmission in Egypt. In children, day care centers contribute to HAV spread among young children, siblings and parents of daycare center attendees.^10^,^11^,^12^

Generally, HAV is acquired early in life with most infections occurring between 5–15 years with no sexual predilection. However, it is unknown what proportions of infected persons are aware of their infection given acute HAV tends to be asymptomatic particularly in children <5 years of age.^10^,^13^ Thus, many individuals are neither tested nor reported. Individuals living in rural areas are at higher risk of hepatitis A infection compared to the urban population due to overcrowding, poor sanitation, certain social practices, and lack of a reliable clean water resource.^12^ A study showed that in Egypt, HAV could be detected in 72%, 50% and 43% of raw sewage, Nile and treated effluent sewage samples respectively.^14^. posing a potential health risk for people.

Within the Egyptian socioeconomic framework and social class structure, differing frequencies of hepatitis A virus infection with different age peaks are observed. In rural or semi-urban regions and lower socioeconomic classes, most of the population is exposed to HAV at a very young age, acquires the infection with minimal or no symptoms and develops immunity.^12^,^13^ Cross-sectional studies conducted in rural areas revealed very high prevalence of anti-HAV IgG reaching 100% of adults.^13^ Consumption of common village water, use of indoor dry pits, and contamination of drinking water with sewage represent the major risk factors for HAV acquisition in rural Egypt.^16^,^17^ In the past 2 decades, there has been significant emphasis on improving sanitation measures and hygiene in Egypt coupled with extension of municipal potable water, sewage, and solid-waste management systems projects. Therefore, in large Egyptian cities and among high social classes, children are infrequently exposed to hepatitis A virus at a young age with subsequent decline in herd immunity and a change to the epidemiology of the illness with steady increases in the mean age of occurrence of illness attributed to acute hepatitis A virus infection. Thus, in urban Egypt despite the overall decrease in the incidence of HAV infection, more HAV infections are encountered at older age groups.

HAV infection in children and adolescents is mostly asymptomatic or might present as flu-like illness or anicteric hepatitis. Symptomatic cases with jaundice that terminates in complete resolution are less common in young age groups. However, in spite of this jaundice, the patient feels well. A proportion of patients who acquire infection at older may develop symptomatic HAV infection with prolonged clinical course and jaundice or may have recurrence within a short period of improvement.^5^,^11^,^12^ Fulminant hepatitis due to HAV is very rare in Egypt although recently few fulminant HAV cases that required liver transplantation have been reported in adult patients who acquired the infection at older age.

Currently, immunization for HAV is not included within the compulsory children immunizations programs in Egypt due to the limited clinical seriousness of the infection among the general Egyptian population.^11^ Immunization is reserved to special groups, namely household/close contacts of patients with HAV, patients with chronic liver disease or HCV infection. Newborns and children may be immunized depending on epidemiology.

Recently several outbreaks of HAV have been reported among European travelers and European tourists returning from Egypt. A survey conducted in 13 European countries traced the source of the outbreaks to food or drinks in hotels or Nile cruises. The reported hepatitis A attack rates varied between tourists from different European countries. Thus, the health authorities in EU countries recommended active vaccination with hepatitis A vaccine for people travelling to Egypt. Adoption of the public health policies and immunization prior to travel are likely to reduce the potential of acquiring HAV infection by non-immune travelers.^6^,^7^,^8^,^9^,^18^

### HAV in Libya:

Similar to Egypt, most HAV infections are acquired during childhood. Surveys showed that HAV antibodies could be detected in 60–70% of three-year old children. By the age of 7years almost 100% of children are HAV immune.^19^

### HAV in Tunisia:

In Tunisia, there is no national program for virological surveillance of hepatitis cases or assessment of epidemiology the of these infections. However, some studies showed that HAV prevalence rates range between 84.0 and 92%.^20^,^21^,^22^ Primary infection with HAV in Tunisia is progressively shifting to older ages, probably due to the improvement of sanitary conditions. A survey conducted to assess the prevalence of HAV among Tunisian children and adolescents revealed an overall seroprevalence among this population was 60% (44%, in children < 10 years old, 58% in those 10–15 years of age, and 83% in those > 15 years of age). Prevalence rates varied according to areas of residence. More than 90% of those living in rural areas had antibodies to HAV compared to 30–50% among those living in the urban areas. The lowest anti-HAV prevalence rates are found in coastal regions probably due to the higher socioeconomic level of the population living in such regions. 20 To date, mass vaccination programs for HAV are not indicated in Tunisia.

Several outbreaks of HAV infection were erupted among European tourists following visits to Tunisia. By age-group, the highest incidence was found in unprotected children travelers 0 to 14 years. Thus, travelers, and especially children, are strongly encouraged to receive HAV vaccination before travelling to South Mediterranean countries including Tunisia.^23^,^24^,^25^

### HAV in Algeria:

Hepatitis A is common in Algeria where 96% of individuals have anti-HAV antibodies. Patients come into contact with the virus before the age of 10. No symptoms are found in 95% of cases while icteric cases are mostly encountered among infants and young children.^26^,^27^,^28^

**Figure:**
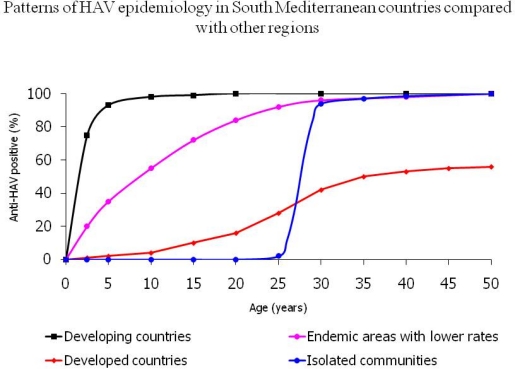


### HAV in Morocco:

Morocco is an intermediate endemic area for HAV infection. The prevalence of anti-HAV antibodies among Moroccan children varies with age. Anti-HAV antibodies could be detected in 45% and 70% in children less than 6 years and children between 7–14 years respectively. Socioeconomic factors, city dwelling and parents education correlated with the prevalence of anti-HAV-IgG. Thus, Morocco is entering a transitional phase with less children exposed to HAV at early childhood. Introduction of hepatitis A vaccination in early childhood might reduce the prevalence of this infection and prevent outbreaks.^29^

## Hepatitis B Virus (HBV) Infection

Hepatitis B virus (HBV) is an ubiquitous virus with a global distribution. Of the approximately 2 billion people who have been infected worldwide, more than 350 million are chronic carriers of HBV.^30^ Approximately 15–40% of infected patients will develop cirrhosis, liver failure, or hepatocellular carcinoma (HCC).^31^ HBV infection accounts for 500.000 to 1.2 million deaths each year and is the 10^th^ leading cause of death worldwide.^30^

South Mediterranean countries have been previously been classified as a high endemicity countries. for HBV.^30^,^31^ Horizontal transmission used to occurs among children and adults. This results in chronic carriage, but less often than when infection occurs vertically or perinatally, leading to a gradual dwindling of the reservoir of chronic carriers with fewer long-term complications and deaths. There is a correlation between the endemicity level of HBV infection and the frequency of HBV related chronic hepatitis and cirrhosis. In fact, where the endemicity level is intermediate or high, HBV infection is common in newborn babies and children and in these groups, progression towards chronicity is frequent. 31,33 On the other hand, late onset of infection such as a result of sexual activity would result in chronic carrier rates of 3±5% become, but 30% may show signs and symptoms of acute infection.

Recent surveys suggest a shift in the HBV endemicity patterns in most Southern Mediterranean countries from high endemicity towards intermediate or low endemicity patterns^33^ with some limited hyperendemic pockets.^30^ Since the early nineties of the last century, Tunisia, Morocco, Libya and Egypt adopted highly effective infants’ vaccination programs which were sometimes extended to the several paediatric and adult groups, producing a dramatic decrease in the incidence of HBV in birth cohorts born after implementation of the vaccination programs. . Furthermore, the increases in the public awareness and modes of transmission of HBV in addition to the improvements in healthcare infrastructure and the socioeconomic conditions contributed to reduce the infection rate in the region.

HBV is divided into eight genotypes (A-H) according to overall nucleotide sequence variation of the genome. The genotypes have a distinct geographical distribution and are used in tracing the evolution and transmission of the virus. Genotype D in is prevalent in North African countries. Differences between genotypes affect the disease severity, course and likelihood of complications, and response to treatment and possibly vaccination.^34^,^35^

### HBV in Egypt:

The incidence and prevalence of HBV showed recent decline following the effective HBV immunization program. In 1985, a study^36^ conducted on 1,866 apparently healthy Egyptians from Upper and Lower Egypt showed that the overall prevalence rate of HBsAg was 10.1%. with higher rates in Upper Egypt (11.7%) compared to the Nile Delta (8.0%). HBsAg was more frequent in young males than females and the prevalence gradually increased with age. A recent study^37^ conducted 20 years later screened 55,922 potentially healthy asymptomatic blood donors for HBsAg. The cumulative seroprevalence of HBV infection was 1.3% with decline in the annual seroprevalence throughout the study period from 2.3% to 0.9%. Another study 13 that compared the frequencies of hepatitis viruses as etiological agents for acute viral hepatitis over the past 2 decades found that the frequency of acute hepatitis B virus infection decreased from 43.4% in 1983 to 28.5% in 2002 (P<.01).

The significant decline in HBV rates indicates the effectiveness of the universal hepatitis B virus immunization of infants that was initiated in 1991. Cross-sectional studies conducted over a decade among schoolchildren in an endemic area in Egypt for screening of HBV virus markers also showed a significant decrease of HBV prevalence among school children confirming the effectiveness of the immunization campaigns.^38^ To evaluate the protective efficacy of hepatitis B vaccine, 720 children aged 10 years who were vaccinated in infancy were tested for hepatitis B serologic markers. Among the vaccinated children, 37.9% had protective anti-HBs, 0.6% had HBsAg while HBV infection occurred in 6.8% of the vaccinated children and it induced a boosting effect on anti-HBs level. only of the vaccinated children.^39^ Despite the decline in the incidence and prevalence of HBV in Egypt, higher HBV prevalence rates are encountered in special patient groups such as patients undergoing hemodialysis 40, patients with hematological malignancies 41, hemophiliacs and injecting drug users (IDUs).^42^

Adults particularly from Upper Egypt had higher prevalence of HBV. In Egypt, various risk factors for HBV transmission were identified including shaving at barbers shops, injecting drug use, recent (<1 year) marriage, home deliveries, occupational or nosocomial exposure in health care facilities.^13^,^43^

In Egypt, HBV genotype D is the predominant HBV genotype (37.1%) followed by genotype B that constituted 25.7%. and mixed D & B infections in 15.7% of patients. HBV genotypes A and C infections were the less observed constituting 10% and 8.6% respectively of the total infections. In children with pediatric malignancies, a relatively high prevalence of mixed A/D genotype infections.^41^

The association of HBV and liver cancer is well documented. The burden of hepatocellular carcinoma (HCC) has increased in Egypt with a doubling in the incidence rate in the past 10 years. The prevalence rates for HBV and HCV were 25.9% and 78.5% among HCC cases. Among HCC cases, HBV significantly decreased over time (p=0.001) while HCV did not, suggesting a shift in the relative influences of these viruses in HCC etiology in Egypt.^44^

### HBV in Libya:

Libya is considered a country with intermediate endemicity for HBV. The seroprevalence of HBsAg among general population in Libya ranges between 5.8% and 1.3% depending on the method of diagnosis and the geographic region. 45,46 However, recent surveys reported an over all prevalence of 2.2% with 120.000–150.000 chronic HBsAg carriers in Libya associated with viral transmission. 47 HBV infection is related to male gender, use of skin scarifications, high-risk behaviors, unsafe injections, history of blood transfusion, family history of hepatitis B or contact with hepatitis B patients. Vertical transmission is responsible for some cases of neonatal HBV infections. In Libya, universal newborns hepatitis B vaccine was introduced since 1993. The vaccine was also recommended to be used to household contacts of HBV-infected individuals and healthcare workers. 47

HBV genotype D is the prevalent HBV genotype as in other countries in North Africa. HBV infection is responsible for the majority of chronic liver disease cases in the country. A high prevalence rate of HBeAg-negative/anti-Hbe-positive chronic hepatitis B has been reported, which accounted for 80% of chronic HBV patients. HBV is a leading cause of hepatocellular carcinoma in Libya.^46^,^47^

### HBV in Tunisia:

Tunisia is considered a country with intermediate endemicity of HBV infection. Many infections are clustered among children and teenagers. Geographically, HBsAg seropositivity varied throughout the country, ranging from 3% to 13% with higher prevalence in the south and central-west regions. Studies conducted in the eighties and nineties showed high overall seroprevalence of HBV in Tunisia reaching 37.5%. 48. However, a more recent survey estimated the overall hepatitis B carrier rates in the general Tunisian population at 3% to7% suggesting a decline in HBV in Tunisia. This could be attributed to the hepatitis B vaccination which was incorporated in the national schedule of new-born vaccination in 1995. 52

The magnitude of vertical and perinatal transmission of HBV is not clear but has been addressed in a study conducted on a cohort of 2303 Tunisian pregnant women of whom 4% were HBsAg positive and 1.4% were vaccinated previously against hepatitis B. Analysis of the risk factors revealed association between the HBsAg status and presence of intrafamilial hepatitis cases (p<0.05). The maternal DNA levels ranged from 34 to 10^8^copies/ml to 10 ^4^ copies/ml. Vertical and perinatal transmission of HBV in the first 3 months of life occurred in 0.4% of 177 mother and child pairs. HBV seroprevalence was 10.7% in infants under 5 years old and increased with age rapidly till 25 years of age and then more slowly in adulthood, reaching 54% for people aged over 40 years. 51

HBV genotypes, (D, A, and E) have been detected in Tunisia with prevalence rates 80%, 8%, and 9%, respectively indicating that genotype D is the most prevalent HBV genotype.^50^ A novel subgenotype, D7, was the most common subgenotype found in asymptomatic Tunisian HBsAg carriers. 53. A high frequency of HBV precore mutants has been reported from Tunisia.^49^ Precore mutants have beeb more frequently found in chronically and in acutely infected individuals, in patients with severe and asymptomatic infections, in HBeAg positive as well as HBeAg negative individuals. 50

### HBV in Algeria:

Algeria is considered a country with intermediate endemicity for HBV. A survey was conducted to assess the serologic markers of hepatitis B (HBsAg, anti-HBc in 1,112 apparently healthy blood donors and 715 pregnant women in different regions in Algeria. HBs Ag was detected in 3.6 % of blood donors and 1.6 % of pregnant women while anti-HBc antibodies in 13 % of blood donors and 11.1 % of pregnant women.^54^ Another Algerian study evaluated the characteristics of hepatitis B viral strains in chronic carrier patients from North-East Algeria. The median age of patients was 35 years and 80% of them had normal transaminase level. Liver histology revealed different necroinflammatory changes in 63% of the patients and established cirrhosis in 21%. “Precore” mutant serological profile without HBe Ag was detected in 87% of patients. Genotype D was predominant (93%) followed by genotype A (5%) and E for one patient. Algerian strains clustered independently from other genotype D reference sequences, suggesting a possible new D subtype.^55^

### HBV in Morocco:

Morocco is among the countries with intermediate/low endemicity of HBV infection. To date, there is no concrete figures for overall prevalence rates of HBV in Morocco. However, some studies showed that 2% of chronic haemodialysis patients treated in a haemodialysis unit in Casablanca show evidence of HBV infection. 58 Genotype D is the most prevalent HBV genotype (97.5%) followed by genotype A, a pattern which is consistent with the predominance of genotype D in the Mediterranean basin. 56 Phylogenetic analysis based on pre-S/S sequences revealed that genotype D in Morocco differed from others D strains subgenotypes (D1, D2, D3 and D4). Pre-core mutant are highly prevalent in Morocco as a study showed that HBeAg-negative/anti-HBe-positive and HBV DNA positive was detected in 86% of 91 patients with chronic hepatitis B. 57

## Hepatitis C Virus (HCV) Infection

Hepatitis C virus (HCV) is a major public health problem and the primary causative agent of chronic liver disease in Southern Mediterranean countries. Compared to other hepatitis viruses, HCV shows vast variations across south Mediterranean countries. Geographically, HCV prevalence rates decrease along the Mediterranean coast from the East towards the West. Egypt has the highest HCV prevalence worldwide with 13% of Egyptians are infected while the prevalence of HCV in Morocco and Algeria is 1–2%. The incidence of HCV in the individual countries is hard to estimate accurately given that many patients with acute HCV infection are asymptomatic. The risk factors and modes of transmission differ among the 5 south Mediterranean countries. HCV genotypes distribution also differ from prevalence of HCV G4 in Egypt and the predominance of genotype 1a or 1b in western areas such as Tunisia and Morocco

### HCV in Egypt:

Egypt has the highest prevalence of HCV in the world (13%). 59,60 The current sequence diversity and phylogenetic tree structure of HCV G4a in Egypt is compatible with the introduction of HCV into that population through parenteral treatment for schistosomiasis with non disposable and poorly sterilized needles in the 1950s and 1960s. 62,63 Although subtype 4a is the dominant Egyptian HCV strain, recent studies revealed that other subtypes are also present, indicating that HCV G4 in Egypt is extremely variable. 64,65,66 HCV accounts for 31% of acute viral hepatitis cases in Egypt. ^676^ In Egypt, high rates of infection are observed in all age groups including young individuals, indicating an ongoing high risk for acquiring HCV infection. More than 60% of acute HCV infections are in persons below the age of 25 yr. ^,^^68^,^69^ High incidence rates (14.1 per 1,000 person-years) have been detected in Egyptian children younger than 10 yr of age living in households with an anti-HCV-positive.^68^ This high incidence in young persons could lead to future increases in chronic disease in these individuals and persistence of the high magnitude of the burden of HCV-related chronic disease in Egypt. The prevalence of HCV in both genders is not well documented although some studies showed higher HCV incidence among men and high spontaneous resolution n rates in women.^69^

In Egypt, relatively higher rates of sexual transmission in Egypt have been reported and reflect the higher background prevalence in this country. In rural Egypt, sexual transmission between monogamous spouses ranged between 3% and 34% (95% CI 0–49). 70,71 Acute HCV is associated with a high temporal risk of transmission of HCV to sexual partners. Sexual transmission, confirmed by phylogenetic analysis, was detected in 15% of sexual partners of individuals with acute HCV. 71 Although some cases of acute HCV have been related to sexual transmission, the degree to which sexual transmission of HCV occurs is controversial because sexual transmission is difficult to confirm given that partners might have other risk factors for HCV transmission such as IDU. Phylogenetic analysis to identify genetic relatedness of HCV viral isolates in partners is required to confirm sexual transmission. All studies on the sexual transmission of hepatitis C are limited by the potential of the confounding variable of IDU or shared items such as razors and other items among sexual partners. 72

HCV infection has been associated in Egypt with health-care-related procedures performed by traditional healers and folk medicine providers, acupuncture, tattooing, body piercing, and commercial barbering. Informal health-care providers and traditional healers perform services that may be associated with HCV transmission such as injections, dentistry, wound treatment, circumcision, excision, and scarification. 73,74 In rural Egypt, about 50% of deliveries are attended by traditional birth assistants. 74 Lack of appropriate cleaning and disinfection of equipment used in these procedures contribute to HCV transmission and the emergence of new HCV cases.^71^

Infection with HCV is an important occupational hazard for Egyptian health-care workers. The risk of occupational HCV transmission increases with deep injuries and after procedures involving hollow-bore needles. In Egypt, occupational transmission among health-care workers through needlesticks and sharps injuries contributes to new HCV cases given that needlestick-prevention devices are not yet adopted most hospitals and health-care units in addition to inadequate compliance with universal, standard precautions in some health facilities. 75,77,78

The role of intrafamilial HCV transmission is still controversial. However, several studies reported HCV spread in families with HCV-infected index cases. In rural Egypt, intrafamilial HCV transmission is considered an important route of transmission where living in a house with an infected family member is a risk factor for HCV transmission. Analysis of data collected during surveys of Egyptian rural communities show that children whose parents had antibodies to HCV were at higher risk for contracting anti-HCV than children whose parents did not.^75^

### HCV in Libya:

In 1994, a study that tested 266 healthy Libyan subjects for anti-HCV antibodies found that 21 (7.9%) subjects had evidence of HCV infection suggesting a high frequency of ‘community-acquired’ HCV in the normal Libyan population. 79 However, the national sero-epidemiological survey in 2006 showed that the prevalence of HCV antibodies in the general population in Libya was 1.2%. 80 In a Tripoli, screening of sera collected over a 2 year period showed that the prevalence HCV of 1.6%, 1.2%, 2% among the general population, blood donors, hospital health care workers respectively. HCV prevalence was 20.5% among renal dialysis patients and 10.8% in the multiple blood transfusion group. 81 History of hospitalization and/or surgical procedures risk (33%), history of blood transfusion. (22.7%), past history of intravenous drug abuse (IVDA) (15%) and history of dental procedures. (15.9%) 81 or medical waste handling 82 were important risk factors for HCV infection. It is not clear whether the discrepancy in prevalence rates between the 1994 report and the 2006 survey report is due to actual reduction or changes in the sensitivity and specificity of the diagnostic tests used.

**Figure:**
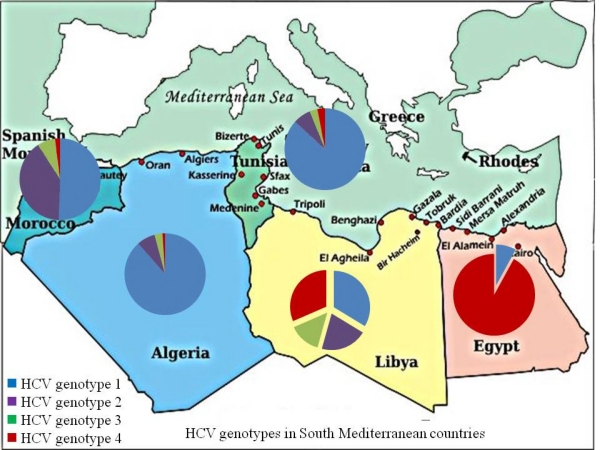


The largest documented outbreak of nosocomial HIV transmission to approximately 400 Libyan children in 1998 was coupled by HCV and/or HBV transmission. 83 Among the HIV infected children, 47% and 33% had HCV and HBV coinfection respectively. Vertical transmission was ruled out by analysis of parents’ serology. Prospective follow up of 160 HIV/HCV coinfected showed that half of the children progressed to moderate or severe immunosuppression and/or moderate or severe clinical symptoms three years after infection. In those who progressed during follow-up, 85% had done so within three years of infection. Children progressing to moderate or severe immunosuppression and/or clinical symptoms were significantly more likely to be receiving ART. 83

The major HCV genotypes circulating in Libya include genotype 1 (G1) in 30.9% of cases followed by G4 (29.2%). Genotype 2 in 19.3% and G3 in 13.6%. The frequency of HCV genotype 4 in the Libya might suggest introduction from neighboring countries mostly from Egypt and Central African countries, where genotype 4 is predominant. Few of the Libyan isolates were closely related to the European ones where the majority of patients are infected with genotype 1, 2 or 3.^83^

### HCV in Tunisia:

The seroprevalence of HCV in the Tunisian general population is low (0.4%). 48 However, another study showed high prevalence of HCV infection among Tunisian dialysis patients with rates of 51% suggesting that the spread may be nosocomial rather than transfusion-related. 85 HCV isolates from 93 Tunisians, including 16 haemophiliacs, were genotyped In non-haemophiliacs, subtype 1b was largely predominant (79%), types 1a, 2a, 2b, 3a and 4a occurred much less frequently at 5, 7, 3, 3 and 1% of cases, respectively. In the group of haemophiliacs, acodominance between subtypes 1a and 1b was noticed (38%). 85

### HCV in Algeria:

The prevalence of HCV in Algeria is not high. In a 1995 survey of 1,112 apparently healthy blood donors and 715 pregnant women in Algeria 54, anti-HCV antibodies were detected in 0.18 % of blood donors and 0.19 % of pregnant women. Recently, the Algerian Ministry of health estimates the prevalence of HCV in Algeria to be around 2.5%.^86^

Occupational exposure and health related transmission seem important risk factors for HCV transmission in Algeria. A survey on the frequency of occupational exposure to HCV among health care personnel in an Algeria hospital revealed 108 exposures in 2 years. Needle stick injuries represented 81% of cases. Source patient serology was unknown in most of the cases, negative in 9% of cases and positive in 10% of cases. 62% of exposed health workers received immediate serology, follow up and screening as of the first day of exposure, 12% after 3 months and 36% after 6 months. No seroconversion case was noted. Cleaning staff and hygiene workers are at high risk of blood contamination as well as nurses, and more than one-third of injuries occurred because of mismanagement of healthcare waste produced in the hospital environment, where needles were not disposed of appropriately in a hard container. Thus, 41.66% of injuries could be avoided if objects were thrown away correctly in specific containers. It is urgent to raise awareness of health care personnel and strengthen adherence to standard precautions as well as to provide suitable containers for the collection and disposal of needles and sharp objects.^87^

### HCV in Morocco:

HCV prevalence rate in Morocco is estimated to be 1.93 %.^88^ However, higher HCV prevalence rates reaching 35.1 and 42.4% are detected among special populations such as patients undergoing haemodialysis and haemophiliacs respectively.^89^,^90^,^91^

The prevalent HCV genotypes in Morroco are 1b (47.6%), 2a/2c (37.1%) and 1a (2.8%). Interestingly, the HCV genotypes distribution varies with age as genotype 1b is more prevalent among older patients, whereas subtype 2a/2c is mainly found among younger ones. 88 Although Morocco belongs to the African continent, the circulating HCV strains are similar to those observed in Europe.

## Hepatitis D Virus (HDV) Infection

Chronic hepatitis D virus (HDV) infection is still an important health problem in the Mediterranean basin region such as in Italy where coinfection with HDV was reported in 9.7%.^92^ Hepatitis D virus (HDV) is an RNA virus that is structurally unrelated to hepatitis A, B, or C virus. It was. HDV causes a unique infection that requires the assistance of viral particles from HBV to replicate and infect other hepatocytes. Its clinical course is varied and ranges from acute self-limited infection to acute fulminant liver failure. Chronic liver infection can lead to end-stage liver disease and associated complications. 92

Approximately 15 million people are infected worldwide. North Africa and the Middle East are considered regions that high prevalence of HDV infections. HDV infection is not associated with a sex predilection. HDV infection is more common among adults than children. However, children from underdeveloped, HDV-endemic countries are more likely to contract HDV infection through breaks in the skin due to the presence of skin lesions.^93^

### HDV in Egypt:

Hepatitis D virus (HDV) infection among Egyptian HBV carriers and patients has not been adequately evaluated. Earlier studies conducted in 1990, demonstrated HDV antibodies in 16.94% of patients with acute HBV and 23.53% in patients with chronic HBV. 94, 95 A more recent study detected anti-HDV in 20% of HBsAg positive subjects and HDV RNA 60%, The proportion of the patients with liver disease was higher in HBV carriers of anti-HDV positive with HDV RNA than in HBV carriers of anti-HDV positive without HDV RNA (P < 0.05). Phylogenetic analysis based on the sequences in nucleotide position 853–1267 of HDV revealed HDV genotype 92.1% of the samples confirming the prevalence of HBV genotype D and HDV genotype I are most prevalent in Egypt. 96 In a prospective study of 45 consecutively Egyptian children (2–15 years) with chronic hepatitis B, anti-delta antibody (IgG anti-HD) was detected in only four children with an overall prevalence of 8.9% (4/45). A substantial association between delta infection and the state of hepatic illness was detected.^97^

### HDV in Tunisia:

Screening of serum samples from 33,363 healthy people in Tunisia for serological markers of hepatitis B and delta showed an overall seroprevalence for HDV of 0.4%. HDV superinfection occurred later than HBV and increased with age in parallel with HBV. HDV superinfection seems common in Tunisia and occurs in almost 44% of individuals infected with HBV.

### HDV in Morocco:

The incidence of delta infections in Morocco seems to be very low in Morocco. In an attempt understand the pathogenesis of severe hepatitis B and acute HBV fulminanat hepatitis observed in this country. Besides one patient with cirrhosis and positive D Ab, neither D Ag nor D Ab is found,. In contrast with the high endemicity of delta agent reported in other countries of North Africa. 98 Considering the possibility the high frequence of high rate of severe hepatitis B (HB) observed in this country could be the contemporary presence of HDV, the presence of delta antigen (D Ag) and antibodies to delta agent (D Ab) was tested by radio-immuno-assay in the sera from 85 HBs Ag positive patients, pitalized in Casablanca: among them 57 suffered from acute or fulminant HB (12 deceased), 10 from chronic hepatitis and 18 from cirrhosis. Neither D Ag nor D Ab were found, excepted once in a patient with cirrhosis having shown the presence of D Ab.^98^

## Hepatitis E Virus (HEV) Infection

Hepatitis E (HEV), previously known as epidemic non-A, non-B hepatitis, is a positive-sense single-stranded RNA icosahedral virus with a 7.5 kilobase genome. HEV as the prototypic member of the genus *Hepevirus*. 99 HEV has been classified further into four major genotypes (I to IV). Genotype I includes Asian strains from India, Burma, Nepal, China, and and African strains from Chad, Algeria, Tunisia, Morocco, Egypt, and Namibia.^100^

This enterically transmitted virus is prevalent throughout much of the developing world. It is constantly present (endemic) in countries where human waste is allowed to get into drinking water without first being purified. Large outbreaks (epidemics) linked to contaminated waterborne sources have occurred in the Indian subcontinent and South American countries where there is poor sanitation. HEV is also a zoonosis. Anti-HEV antibodies have been detected in rodent, swine, sheep, bovine and poultry. 99, 100

In general, hepatitis E is a short-lived, self-limiting viral infection followed by recovery. Prolonged viraemia or faecal shedding are unusual and chronic infection does not occur. The incidence of hepatitis E is highest in juveniles and adults between the ages of 15 and 40. Though children often contract this infection as well, they less frequently become symptomatic. Mortality rates are generally low. Occasionally, a fulminant form of hepatitis develops, with overall patient population mortality rates ranging between 0.5% - 4.0%. Fulminate hepatitis occurs more frequently in pregnancy and induces a mortality rate of 20% among pregnant women in the 3rd trimester and can also cause premature births.^99^,^100^

The prevalence and endemicity of HEV vary across Southern Mediterranean countries. The features of HEV infection in North Africa is different from that in the Indian subcontinent. Despite the high prevalence rates in southern Mediterranean countries, HEV infection is rarely symptomatic in the region and fulminant hepatitis cases among pregnant women are uncommon. However, given that there is no immunization against HEV, travelers from non-endemic areas to these countries must be aware of the risk of HEV infection and should adhere to strict hygienic measures, such as drinking bottled water and eating cooked food.

### HEV in Egypt:

Hepatitis E virus is probably endemic in Egypt and seems to be a frequent infection. Genotypic characterization of HEV circulating in Egypt showed the predominance of genotype 1 HEV related to other North African isolates is circulating in acute symptomatic patients in Egypt. 101

HEV accounts for 20—40% of adult and pediatric hospitalized cases of acute viral hepatitis. 67,95, 101 Community surveillance revealed a high prevalence of anti-HEV antibodies within several rural populations 102, 103, 104 A cross sectional survey in several villages in Upper Egypt and the Nile Delta revealed anti-HEV antibodies in 67.7% of healthy adults^102^ while screening of Egyptian blood donors in Cairo demonstrated anti-HEV antibodies in 24% of studied subjects.

The seroprevalence of anti-HEV is high among Egyptian children and the rate of positive antibodies increases with age. In the first decade of life, the prevalence of antibodies has been shown to exceed 60% then it peaked to 76% in the second decade and remained around 70% thereafter. HEV serological markers are frequently encountered among children with acute viral hepatitis in conjunction with hepatitis B viruses. Dual infection with HEV and other hepatotropic viruses has been reported in children with great elevation of aspartate and alanine aminotransferases. Among children (1–11 years old), with acute symptomatic hepatitis, HEV was recognized as etiological factor in 22% of cases.^102^

The prevalence of HEV antibodies in pregnant females in rural areas in Egypt is very high. A cross-sectional survey of a cohort of pregnant women living in three rural villages in the Nile Delta demonstrates the high prevalence of anti-HEV that reaches 85%. suggesting universal community exposure to this enterically transmitted virus. Despite reports of fulminant HEV hepatitis during pregnancy in Asia, there is little evidence that HEV-caused overt hepatitis in the pregnant women and there have been no reports of the effects of HEV on pregnant women and their offspring in Egypt. 105

Thus, the prevalence of HEV in Egypt is among the highest reported in the world. Despite the very high prevalence of anti-HEV, HEV epidemics are uncommon in Egypt and. almost no one of the screened subjects gave a prior history of jaundice, hepatitis or liver disease, and none of these symptoms were significantly associated with anti-HEV. This lack of HEV-associated morbidity could be due to the presence of a less virulent circulating strain of HEV in the population (and possibly in all of Egypt); the presence of cross-reacting antibodies to an HEV-like virus which is not associated with clinical disease; or a high prevalence of exposures during the early years, which may be more likely to cause asymptomatic, anicteric illness, as is the case for HAV in endemic regions Exposures to HEV in early childhood may modify responses to subsequent exposures, such that clinical illness due to HEV is rare, as it is in endemic HAV. 101

### HEV in Libya:

Epidemic and sporadic water-related diseases that might be attributes to HEV have been reported in Libya.^19^,^106^ However, no accurate estimates of the prevalence of HEV are available. Transmission of the HEV in Libya might be related to eating raw or uncooked shellfish. Farm animals, may serve as a viral reservoirs as HEV is one of the few viruses which has been shown to be transmitted directly from animals through food

### HEV in Tunisia:

This prevalence of HEV in Tunisia is about 4.3%. 20 No epidemics attributed to HEV have been reported in Tunisia suggesting that the virus might be circulating among the Tunisian population as sporadic cases. Higher anti-HEV IgG prevalence rates reaching 12.1 % have been reported among pregnant women. In multivariate analysis, age (>30 years) and the number of persons per room (>2) in the house were independent factors predicting HEV infection. History of agricultural work, kind of water, sewage treatment, use detergent to wash vegetables, contact with animals and parenteral risk factors were not correlated with the presence of anti-HEV IgG. 107

**Figure:**
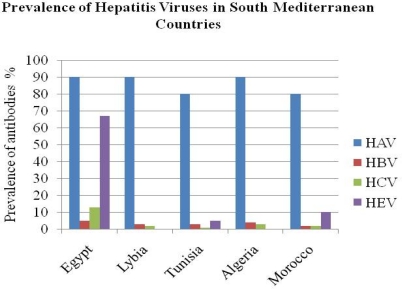


### HEV in Algeria:

Hepatitis E is endemic in Algeria but the accurate ratesare not available. Sporadic cases frequently emerge or may go unrecognized or under-reported. However, several epidemics of hepatitis E have been reported in Algeria such as the HEV) Algerian epidemic between 1978–1980 and the epidemic in Tanefdour, Algeria, in 1986–1987. 108 Before the identification of hepatitis E virus in 1990, such hepatitis outbreaks were denominated enterically-transmitted non-A, non-B hepatitis. In January and February 1987, an outbreak of acute hepatitis occurred in the eastern territories of Algeria. Among the affected individuals, two pregnant women died by fulminant hepatitis. The epidemiological investigation traced the epidemic to a common source water contamination. Genotyping of the isolates showed that all the cases were due to HEV genotype I. Most isolates shared 99.7–100 % sequence identity and the remainder showed 1–1.3 % divergence. Interestingly, intra-patient heterogeneity revealed sequence diversity ranging from 0.11 to 3.4 suggesting a probable quasispecies organization of HEV during epidemics and could explain the adaptable behavior of the virus in the host-pathogen interrelations. Comparing the partial nucleotide sequences and derived peptide sequences of hepatitis E virus (HEV) from two outbreaks of hepatitis E in Algeria and Chad revealed 92 and 95%, homology at the nucleic acid level and 98% the peptide level respectively. 108,109 Thus, the African strains of HEV appear to be a distinct phylogenetic group, separate from the Mexican and Asian strains.

### HEV in Morocco:

HEV is also endemic in Morocco. Among healthy adult Moroccans, anti-HEV IgG has been detected in 6.1%–10.4% of subjects particularly in the west and the south of Morocco. Subclinical HEV infection is frequent in children and young adults. A longitudinal study showed that anti-HEV IgG in healthy contacts decreased significantly after 30 years of age. The incidence of clinical acute HEV infections increased significantly with age.^110^

In 1994, an of acute HEV outbreak erupted in the south of Morocco. HEV was confirmed using recombinant antigen-based enzyme immunoassays and reverse transcription polymerase chain reaction in 77.3% of patients. Furthermore, HEV-specific IgM was positive in 84.0% of cases and was associated with subclinical HEV infection in contacts of index cases. Faecal contamination of drinking water samples collected from the epidemic city was observed. It also appeared that primary infection with HEV accounted for more than 86% of the cases. A longitudinal study showed waning of anti-HEV antibodies in patients and healthy contacts six months after the initial testing. 111

## Conclusions:

Taken together, the epidemiology, frequency and patterns of hepatitis viruses show variations across the Southern Mediterranean region depending on several demographic and socioeconomic factors. The prevalence of viral hepatitis infection is still high and represents a serious public health challenge Hepatitis B and C viruses remain to be the major causes of chronic hepatitis in the region. Hepatitis A and E are endemic in the region and despite the mainly subclinical features of the infections among residents, HAV and HEV represent a risk to tourists and expatriates visiting the Southern Mediterranean countries. Better control of viral hepatitis is required to decrease the burden of the disease. Immunization against hepatitis A virus and hepatitis B virus universal use of disposable syringes and implementation of better hygienic conditions are crucial measures to reduce the impact of viral hepatitis in South Mediterranean countries.

**Table:** Demographic Characteristics in Acute Hepatitis Cases Sentinel Hepatitis Surveillance, Egypt, 2001–2004

	Hepatitis Markers			
	HAV (n=1684)	HBV (n=1256)	HCV (n=1249)	Non A-C (n=1720)
Median Age (years)	9	28	45	33
Rural	4	25	42.5	23
Urban	14	30	46	35
Range of Age	13 m–79 y	2 m–87 y	3 y–90 y	2 m–94 y
% Male	63%	70%	68%	57%
Case fatality ratio	0.2	0.4	0.3	0.2
